# Welding Technology and Heat Treatment Butt-Welded Joints of Thin-Walled Inconel 718 Alloy Tubes

**DOI:** 10.3390/ma18163896

**Published:** 2025-08-20

**Authors:** Patryk Warchoł, Lechosław Tuz

**Affiliations:** Faculty of Materials Engineering and Industrial Computer Science, AGH University of Science and Technology in Krakow, 30-059 Krakow, Poland; warcholpatryk@gmail.com

**Keywords:** orbital welding, nickel alloy, Inconel 718, mechanical properties, microstructure, heat treatment, thin-walled tube

## Abstract

The subject of this research was the development of technology for welding and the heat treatment of butt-welded joints of thin-walled Inconel 718 alloy tubes, a process based on orbital TIG welding without a filler metal. The developed technology allows favorable conditions to obtain the appropriate hardness and mechanical properties in the weld area required by AMS 5589. In the tests, the microstructure and mechanical properties were evaluated. Compliance with the requirements was evaluated on the basis of metallographic and mechanical properties tests. The results obtained indicate that the weakest area of the joint is the base material of the thin-walled tube. The welded joints reveal elongation above 10% and tensile strength above 1400 MPa despite the dendritic structure of the weld. In the area of the welded joint, the occurrence of precipitates of the γ’ phase and mainly niobium carbide was revealed.

## 1. Introduction

Nickel-based alloys, due to their unique properties, are one of the most important groups of engineering materials used in various industries, which, due to their specific characteristics, force the selection of materials capable of operating in extreme environmental conditions, including the aviation, energy, and petrochemical industries [1]. Thanks to the combination of very good mechanical properties and resistance to surface degradation in the high-temperatures range, the components made of nickel alloys are used wherever high heat resistance, the ability to take loads at high temperatures, or good creep resistance are required [2]. Some of the nickel and cobalt superalloys can be used at temperatures as low as 0.85 homologous [3,4].

The unusual properties of nickel alloys are due to their chemical composition and thermomechanical processing at the metallurgical process stage, as well as the stability of the austenitic structure [1,4]. The main types of nickel alloys include precipitation-strengthened alloys and solid solution strengthened alloys [5,6]. Precipitation-strengthened alloys, in particular, have found application in aviation, but due to the increased proportion of elements that form strengthening phases (γ’ and γ”) such as titanium, aluminum, and niobium, these alloys are more difficult to join using the welding process than solid solution strengthened ones [7,8]. The main problems associated with welding of precipitation-strengthened alloys include the following: crystallization cracking, cracking in the HAZ, strain cracking due to aging [9,10], segregation, as well as formation of Laves phases [7]. In addition, along with the content of alloying elements, the problem increases during welding [4]. Based on the studies by [11,12], alloys strengthened with the Ni_3_(Al, Ti) phase are more problematic in terms of the use of the welding process and the joining than alloys strengthened with the Ni_3_Nb phase [8,9]. This is due to the kinetics of the formation of these phases as a result of heat interaction [11,13]. The shape and morphology of precipitates and their arrangement in the structure are mainly influenced by the applied heat treatment [14,15].

Joining technologies are used to join nickel alloys in aerospace, marine ships, power, and other fields [8,13]. Despite the observed problems, research and work on welding technologies is still being carried out. In conventional processes such as arc welding (TIG), the influence of heat input by a direct current [16] or pulse arc [17] are analyzed as technological aspects of joining. On the other hand, the effect of filler metal [18] and changes in the fusion [19] and corrosion behavior [20] are tested. To decrease the size of the weld, and to avoid the use of filler material [21], a high energy laser beam (LBW) can be used [22,23]. To control heat input and fusion depth, beam frequency [24] or beam oscillation [25] can be used. Also, different wavelengths are used [26,27]. The same effect of heat (power) density is observed in the electron beam welding (EBW) [28,29]. The obtained welds, due to different heat concentrations resulting from the power density and heat input, exhibit slightly different mechanical, plastic, or structural properties [30]. The wide application of welding technology in joining materials is the result of the generally accepted good weldability of the solid solution strengthened Inconel 718 alloy and favorable operational properties of the joints. The operational properties are a consequence of the applied heat treatment [31,32].

The TIG orbital welding process is generally designed for the butt welding of tubes (circumferential welds). However, joining thin-walled components is challenging, as discontinuities in the weld seam can occur due to porosity, lack of fusion, or hot cracks. To compensate, the heat input to the material is typically increased, which consequently leads to weld collapse and a reduction in the pipe’s effective cross-section. The thin wall thickness and small diameter require welding to achieve a favorable weld geometry with a slightly convex root and face. This shape ensures proper fluid and gas flow within the pipe and reduces erosion and cavitation. Therefore, in this work, tests were conducted to obtain optimal properties after welding and heat treatment, including solution annealing and alloy aging. This research was carried out for welded tubes from the manufacturing process (not samples) where the orbital welding process of thin-walled pipes was successfully applied, a novelty in aircraft applications, and providing further opportunity for process optimization and automation.

## 2. Materials and Methods

**Figure 1:**
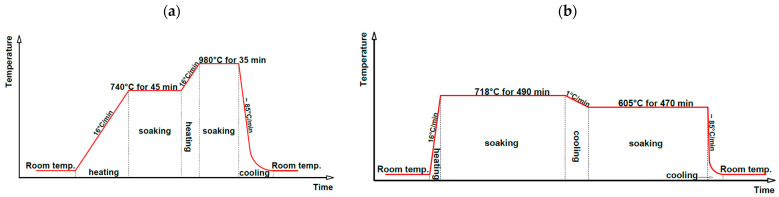
Heat treatment parameters: (**a**) the solution annealing parameters and (**b**) the aging parameters.

The base material was Inconel 718 nickel alloy tubes strengthened with γ” phase particles (100 × 6.35 × 0.7 mm), which were joined by orbital TIG welding (142 to EN ISO 4063 [33]). Welding was performed using an Orbimat 180 SW current source and an Orbiweld 38S closed head (Orbitalum Tools GmbH, Singen, Germany). In safety-critical components or installations requiring high reliability, the use of heterogeneous joints is not permitted. During orbital welding, depending on the electrode’s position relative to the pipe axis and the pipe axis orientation itself, significant property inhomogeneity can occur, which is a consequence of the variable amount of heat introduced into the material. Therefore, this article presents the results after heat treatment, which ensures homogeneity of properties along the entire length of the weld. The resulting butt joints were subjected to post-weld heat treatment for optimal mechanical properties, which was carried out in a vacuum furnace that provided a vacuum of not less than 10^−4^ Tr. Table 1 and Table 2 show the chemical composition and mechanical properties of the material in the as-delivered conditions, as well as the required mechanical properties after the precipitation strengthening. Furthermore, Table 3 and Figure 1 include selected welding and heat treatment parameters to which the joints were subjected. In the test, Argon 4.8 and 5.0 were used.

The joints were evaluated on the basis of visual (VT) testing, according to EN ISO 17637 [34] requirements, and macroscopic and microscopic observations including both light (LM) and scanning electron microscopy (SEM). Analysis of the chemical composition of the precipitates and matrix was carried out using EDS. The mechanical properties of the joints were determined by performing a static uniaxial tensile test and hardness measurements in the cross-section of the joints. The rupture points of the samples were subjected to fractographic analysis.

For macro- and microstructure analysis, the metallographic specimens were made in cross-section of the joints, which were then ground, polished, and subjected to electrolytic etching in CrO_3_. The specimens thus prepared were observed using Leica DM/LM light (Leica, Wetzlar, Germany) microscopy and Phenom XL (Thermo Fisher Scientific, Waltham, MA, USA) and Nova NanoSEM 450 (FEI, Waltham, MA, USA) scanning electron microscopy (SEM). The microhardness tests using the Vickers HV0.3 method covered all areas of the joint and the test was performed on a TUKON 2500 hardness tester (Wilson Instruments, Norwood, MA, USA). Uniaxial tensile testing was carried out using an 810 MTS test machine (MTS Systems Corporation, Eden Prairie, Minnesota, USA), and the tested joints were stretched at a speed of 5 mm/min. The static tensile test was performed on the welded and HT pipes, without additional mechanical treatment. The total length of the samples was 150 mm, with a gauge length of 50 mm, maintaining the weld at its center. To secure the weld in the machine’s handles, a solid rod was inserted into the tube interior to limit flattening of the tube by the handle pressure.

## 3. Results and Discussion

**Figure 2:**
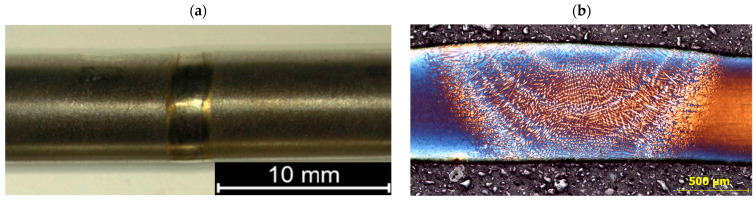
Macrostructure of welded joint: (**a**) general view, (**b**) in cross-section.

VT tests did not reveal surface defects that occur in the rims of the joints, such as underfills, porosity, and overfills. No discontinuities in the material in the form of cracks were also observed. Visual and macroscopic observations showed that all joints met the quality level B according to EN ISO 5817 [35] (Figure 2a).

**Figure 3:**
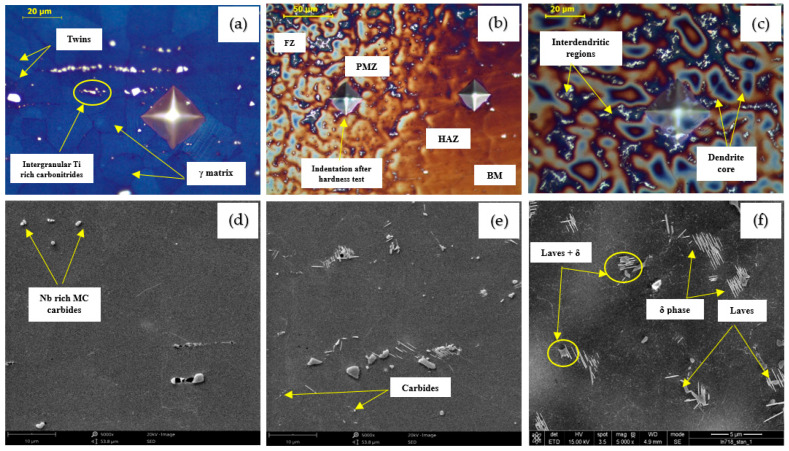
Light microscope (**a**–**c**) and scanning electron microscope (**d**–**f**) images of the microstructure (BM, HAZ, and WM) of Inconel 718 welded joints.

Microscopic examination included all zones of welded joints, i.e., the weld, the heat-affected zone, and the base material (Figure 2b and Figure 3). In addition, observations were made of the material under delivery conditions.

The material after welding and under delivery conditions has a γ phase microstructure, characteristic of nickel alloys. In the structure, a large number of precipitates was revealed, where, depending on the weld zone, the morphology of the precipitates varied (Figure 3d–f). In the base metal of the welded joints, different size globular morphology precipitates of were revealed. The observed precipitates were gold-etching titanium carbides and bright niobium carbides (NbC). Some of these precipitates formed bands resulting from the applied plastic processing of the material performed at the pipe manufacturing stage (Figure 3a). The base material of the joint also revealed a sizable number of annealing twins resulting from the applied post-weld heat treatment. All joints were characterized by a very narrow heat-affected zone resulting directly from the very small amount of heat input (≤0.066 kJ/mm) during the welding process (Figure 3b). In the HAZ, a transitional morphology of precipitate was observed between massive globular precipitates present in the base material and plate-shaped precipitates present in the weld (Figure 3e). All analyzed welds were characterized by the correct shape and structure typical of Inconel 718 welded joints. In this area, the dendritic, crystalline structure was well visible (Figure 3c). Plate-shaped precipitations were located in the interdendritic areas, and in some cases carbide nitrides were also observed. As a result of the etching used, stops of crystallization, symmetrical with respect to the weld axis and formed during the solidification of the liquid pool, were revealed.

Observations using scanning electron microscopy (SEM) confirmed the presence of precipitates in the material under delivered conditions, of which the more massive ones were distributed within the grains, while the finer precipitates were located at grain the boundaries. The precipitates were subjected to EDS analysis, the results of which indicated that the precipitates present in the alloy were complex niobium carbides and titanium carbonitrides. The sizes of the precipitates varied and ranged from about 15 µm for the largest niobium carbides (NbC) to precipitates of less than 1 µm in size. Titanium carbon nitrides were mainly less than 7 µm. Near the fusion line, plate-morphological precipitates were present, while together with moving away from the fusion line toward the base material, globular, massive niobium precipitates accounted for an increasing proportion of all precipitates. A tendency to increase the number of precipitates with a decrease in the distance to the fusion line was observed. Observations with SEM and EDS analysis of intermetallic phases with plate morphology present in the weld indicated the existence of the δ phase (Ni_3_Nb) in the area of which the Laves phase (A_2_B) has formed (Figure 3f) [11,17,18]. Furthermore, very fine precipitates of less than 1 µm in size were revealed from an almost continuous lattice (Figure 3f).

**Figure 4:**
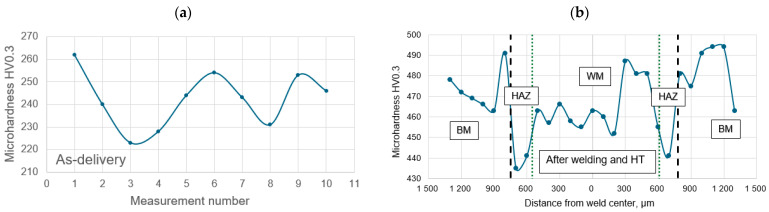
Distributions of the HV0.3 hardness in the material as delivered (**a**) and the cross-section of the joints after heat treatment (**b**); dashed line—range of HAZ after welding, dot line—fusion line, 0—weld center.

Cross-sectional hardness measurements showed an increase in hardness for the material under delivered conditions compared to the welded and heat treatment joints. Distribution and average hardness values are shown in Figure 4 and Table 4. Under delivery conditions, the average hardness was 242 HV0.3. The hardness measured in the joint showed a slight decrease within the HAZ (448 HV0.3) relative to the base material (468 HV0.3) and the weld (460 HV0.3). The results obtained for the tested joints meet the minimum hardness requirements (36 HRC) for Inconel 718 in the hardened state according to AMS 5589. The significantly lower hardness in the HAZ is likely a result of grain coarse near to the fusion line.

**Figure 5:**
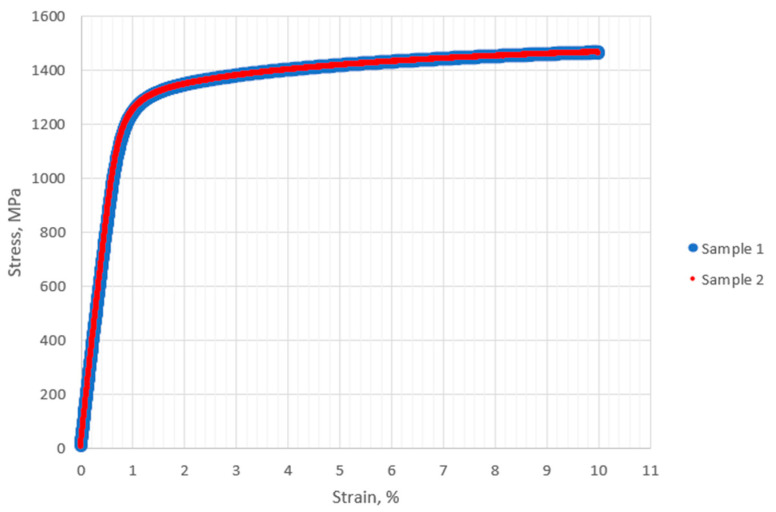
Engineering tensile test curves.

**Figure 6:**
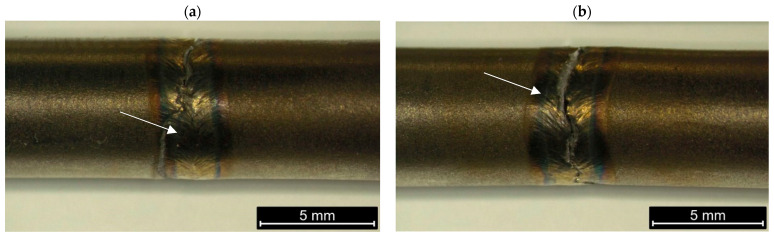
Samples after tensile test. White arrow indicate placement of fracture (**a**,**b**).

Static uniaxial tensile testing was performed on two samples and showed that the joints tested exhibited a tensile strength of approximately 1465 MPa and a yield strength of approximately 1277 MPa (Figure 5). In each case, the crack formation was located within the weld (Figure 6a,b) and the elongation of the samples was 10% (Table 5). The tensile strength and yield strength obtained meet the requirements for seamless Inconel 718 tubes in the hardened state according to AMS 5589.

**Figure 7:**
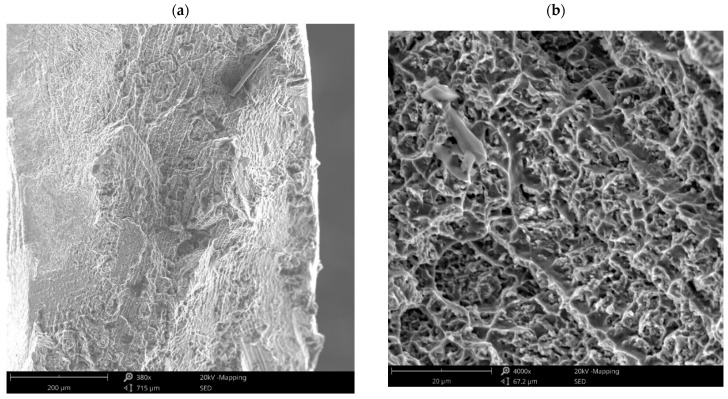
Fractographic images of tensile fracture: (**a**) general view, (**b**) ductile fracture.

Fractographic studies revealed the ductile nature of the fractures (Figure 7a). In some places, the surface of the fracture was drawn at an angle of 45°. In addition, the surface of the breakthroughs revealed precipitates with niobium. A coarse crystalline structure of the weld was observed with visible outlines of crystallites and dendrites (Figure 7b). In addition, hot cracks and microporosity formed in the weld metal during crystallization were revealed. EDS analysis of the globular precipitations (about 10 µm) observed at the fracture surface indicated that it is a slag inclusion. The fracture occurred in a single, privileged plane. Such a phenomenon was favored by the decoration of the grain boundary, which results in easier shearing of such material.

The technological tests and microscopic examinations performed on Inconel 718 orbital welded joints showed that, with the use of the given welding parameters and heat treatment, it is possible to produce joints that meet the requirements of the quality level B according to EN ISO 5817 [35]. The joints after the conducted precipitation hardening met the requirements of AMS 5589 in terms of mechanical properties such as hardness, tensile strength, and yield strength. Moreover, the studies performed indicate that the use of heat treatment including solution heat treatment and annealing is a beneficial way to obtain the required mechanical and plastic properties. Additionally, it should be noted that despite the subsequent solution heat treatment process, i.e., first, the tubes themselves, and in tests of tubes with welded joints, it did not cause unfavorable changes and caused the appearance of additional precipitations.

## 4. Conclusions

The tests carried out revealed the following:

- Correct shape and geometry of the fabricated joints, characterized by the high quality of the face and root;
- Symmetrical fusion zone with a very narrow heat-affected zone. In the various zones of the joint, a typical microstructure of welded joints made of Inconel 718 was observed. The alloy matrix consisted of a γ phase of the FFC structure and precipitations of niobium carbides (NbC) and titanium carbide in it. No microcracks were observed in the HAZ area; however, a change in the morphology of the precipitates from globular to lamellar was evident in this zone. The welds were characterized by a dendritic structure. Thanks to the EDS analysis, a tendency of the niobium was observed to segregate in the interdendritic areas, where there were δ phase (Ni_3_Nb) with lamellar morphology and Laves phase;
- An increase in hardness after the applied precipitation hardened heat treatment relative to the material under delivery conditions from 240 HV0.3 to 468 HV0.3. The measured microhardness (HV0.3) has met the requirements according to AMS 5589. In all zones of the joints, the hardness was similar (±10 HV0.3);
- The tensile strength (1464 MPa) and yield strength (1277 MPa) met the requirements for welded joints according to AMS 5589. The placement of the fracture was the weld area, which is typical for a girth weld. This placement of crack means that, despite the use of favorable heat treatment, the fatigue resistance of the joints will be limited;
- The ductile fracture of the resulting fractures was observed, on the surface of which a large number of precipitates were observed as well as the inclusion of slags, gas micropores formed during crystallization, and small hot cracks. Despite the local discontinuities, the fractures studied can be assessed as correct.

## Figures and Tables

**Table 1:** Chemical composition of Inconel 718 (AMS 5589), wt.%.

Wt.%	C	Mn	Si	Cr	Ni	Mo	Nb	Ti	Al	Co	Ta	B	Cu
Min	0	0	0	17	50	2.8	4.75	0.65	0.2	0	0	0	0
Max	0.08	0.35	0.35	21	55	3.3	5.5	1.15	0.8	1.0	0.02	0.006	0.3

Note: Fe—balance; P and S—max 0.015.

**Table 2:** Mechanical properties of Inconel 718 tubes in delivery state and required after heat treatment.

Mechanical Properties	Delivery Conditions	After Precipitation Heat Treatment
Tensile strength, max	1069 MPa	1276 MPa
Yield strength, max	655 MPa	1034 MPa
Elongation 50 mm, min	30%	12%

**Table 3:** Orbital TIG welding process parameters.

Parameters	Sectors				
	1	2	3	4	5
Welding current HP [A]	18.2	17.6	16.7	15.4	14.5
Welding current LP [A]	6.3	6.1	5.8	5.4	5.0
Welding average voltage [V]	10.0	10.0	10.0	10.0	10.0
Travel speed HP [mm/min]	100	100	100	100.0	100
Travel speed LP [mm/min]	100	100	100	100	100
Argon flow [l/min]	5.6	6.0	6.0	6.0	6.0

**Table 4:** The average values of the hardness measurements [HV0.3].

Condition	Base Metal	Heat-Affected Zone	Weld Metal
Delivery conditions	242	-	-
After welding and heat treatment	478	439	465

**Table 5:** Tensile tests results.

Sample No.	Fracture Locations	Tensile Strength [MPa]	Yield Strength [MPa]	Elongation [%]
1	Weld metal	1465	1278	10
2	Weld metal	1464	1277	10

## Data Availability

The original contributions presented in this study are included in the article. Further inquiries can be directed to the corresponding author.
